# Lemur Tyrosine Kinase 2, a novel target in prostate cancer therapy

**DOI:** 10.18632/oncotarget.3899

**Published:** 2015-05-08

**Authors:** Kalpit Shah, Neil A. Bradbury

**Affiliations:** ^1^ Department of Physiology and Biophysics, Rosalind Franklin University of Medicine & Sciences, The Chicago Medical School, North Chicago, IL 60064, USA

**Keywords:** LMTK2, androgen receptor, castrate resistant prostate cancer, prostate cancer, kinases

## Abstract

Progression from early forms of prostate cancer to castration-resistant disease is associated with an increase in signal transduction activity. The majority of castration-resistance cancers persist in the expression of the androgen receptor (AR), as well as androgen-dependent genes. The AR is regulated not only by it associated steroid hormone, but also by manifold regulatory and signaling molecules, including several kinases. We undertook evaluation of the role of Lemur Tyrosine Kinase 2 (*LMTK2*) in modulating AR activity, as several Genome Wide Association Studies (GWAS) have shown a marked association of *LMTK2* activity with the development of prostate cancer. We confirm that not only is *LMTK2* mRNA reduced in prostate cancer tissue, but also *LMTK2* protein levels are markedly diminished. Knockdown of *LMTK2* protein in prostate cell lines greatly increased the transcription of androgen-responsive genes. In addition, *LMTK2* knockdown led to an increase in prostate cancer stem cell populations in LNCaP cells, indicative of increased tumorogenicity. Using multiple approaches, we also demonstrate that *LMTK2* interacts with the AR, thus putting *LMTK2* as a component of a signaling complex modulating AR activity. Our finding that *LMTK2* is a negative regulator of AR activity defines a novel cellular pathway for activation of AR-responsive genes in castrate resistant-prostate cancer. Moreover, pharmacologic manipulation of *LMTK2* activity will provide a novel therapeutic target for more effective treatments for patients with castrate-resistant prostate cancer.

## INTRODUCTION

The androgen receptor (AR), a ligand-dependent nuclear receptor, plays a critical role in prenatal development of the prostate [1–3]. For example, work by wilkins and colleagues in 1950 showed that individuals with complete androgen insensitivity, caused by AR inactivating mutations, do not develop a prostate gland. Likewise, AR knockout mice show a lack of prostate gland development [4]. Even in adult males, the AR continues to support the survival of secretory prostate epithelia, the primary cell type argued to be transformed in prostate adenocarcinoma [5, 6]. Prostate cancer is the second leading cause of cancer related death amongst men in the united states, with some 233,000 individuals diagnosed with prostate cancer in 2014 alone, of which ~30,000 died as a consequence of the disease [7]. Many of the patients who develop prostate cancer receive AR antagonists, to inhibit the actions of androgens on proliferation of prostate epithelia [8, 9]. These therapies although effective in initial stages, quickly loose their benefit, as most patients eventually develop a castrate resistant prostate cancer (CRPC), a devastating though poorly understood disease state [10–13]. Studies using xenograft prostate tumors that recur following androgen ablation therapy show that although CRPC is insensitive to further androgen depletion treatment, the tissue still expresses AR regulated genes, suggesting that AR signaling pathways are still intact in CRPC [14, 15].

Kinase-signaling pathways have been implicated in the regulation and modulation of nuclear receptor activity [16, 17], [18–20]. However, the signaling mechanisms through which kinases modulate AR function are not well understood. Stress kinase signaling is known to regulate AR phosphorylation, increasing its rate of nuclear export and proteasome-mediated degradation [21]. In contrast, Protein Phosphatase 1C (*PP1C*) dephosphorylates AR, leading to an increase in AR nuclear retention and increased gene transcription [22]. Furthermore, the HER2/ERBB3 kinase signal has been shown to stabilize AR protein levels and optimize binding of the AR to promoter regions of androgen-regulated genes [23]. Clearly, dysregulation of kinase signaling pathways would impact heavily on AR activity in CRPC [24].

**Figure 1:**
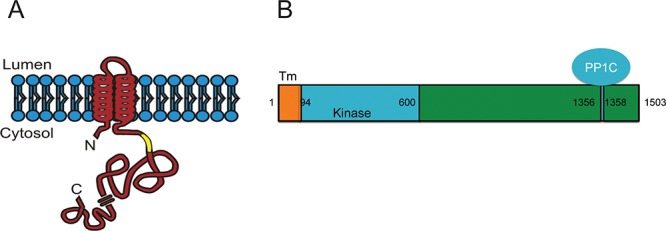
Predicted structure of Lemur Tyrosine Kinase 2 (*LMTK2*) **A.** Topology of *LMTK2* in endosomal membrane is shown with N-terminal and C-terminal either in cytosol or within an endosomal lumen [26]. **B.** *LMTK2* is 1503 amino acid long protein with predicted transmembrane (TM) helices located between 11–29 and 46–63 amino acids while kinase domain is predicted to lie between 94–600 amino acid as shown in yellow. *LMTK2* interacts with Protein Phosphatase 1 C (*PP1C*) via its VTF motif (1356–1358 amino acids) [25].

Recent Genome Wide Association Studies (GWAS) of patients with prostate cancer have identified a genetic variant of Lemur Tyrosine Kinase 2 (*LMTK2*) (also called BREK/KPI-2/CRPK/AATYK2), a membrane associated kinase (Fig. 1A) [25, 26], [27, 28] to be strongly associated with prostate cancer (*P* < 0.0001) [29–32]. Furthermore, this genetic variant of *LMTK2* has a Single Nucleotide Polymorphism (SNP) in intron 9, causing decrease in *LMTK2* mRNA levels [29]. These studies suggest that *LMTK2* might be involved in the development and/or maintenance of prostate gland tumors. However, due to limited understanding of *LMTK2* function [33, 34], its role in prostate cancer still remains unknown. Recently, *LMTK2* has been reported to interact with *PP1C* (Fig. 1B) and inhibit its activity in CNS [35, 36]. Since, *PP1C* plays an important role in nuclear retention of AR by dephosphorylating AR, it is likely that decreased *LMTK2* protein and/or activity would result in an increase in AR activity and sensitivity to androgens, events precisely observed in CRPC.

In summary, the results in this manuscript argue that *LMTK2* interacts directly with AR and negatively regulates its activity. Furthermore, a decrease in *LMTK2* protein expression, as proposed in prostate cancer, not only results in an increase in androgen mediated AR activity but also increases the androgen-independent activity of AR. Moreover, *LMTK2*-Knock Down (KD) in prostate cancer cells results in an increase in cell viability and tumorogenecity in the presence and absence of androgen. As such, our study takes observations made in genomic studies and reveals *LMTK2* as a novel regulator of AR in prostate epithelium.

## RESULTS

### *LMTK2* expression and localization

**Figure 2:**
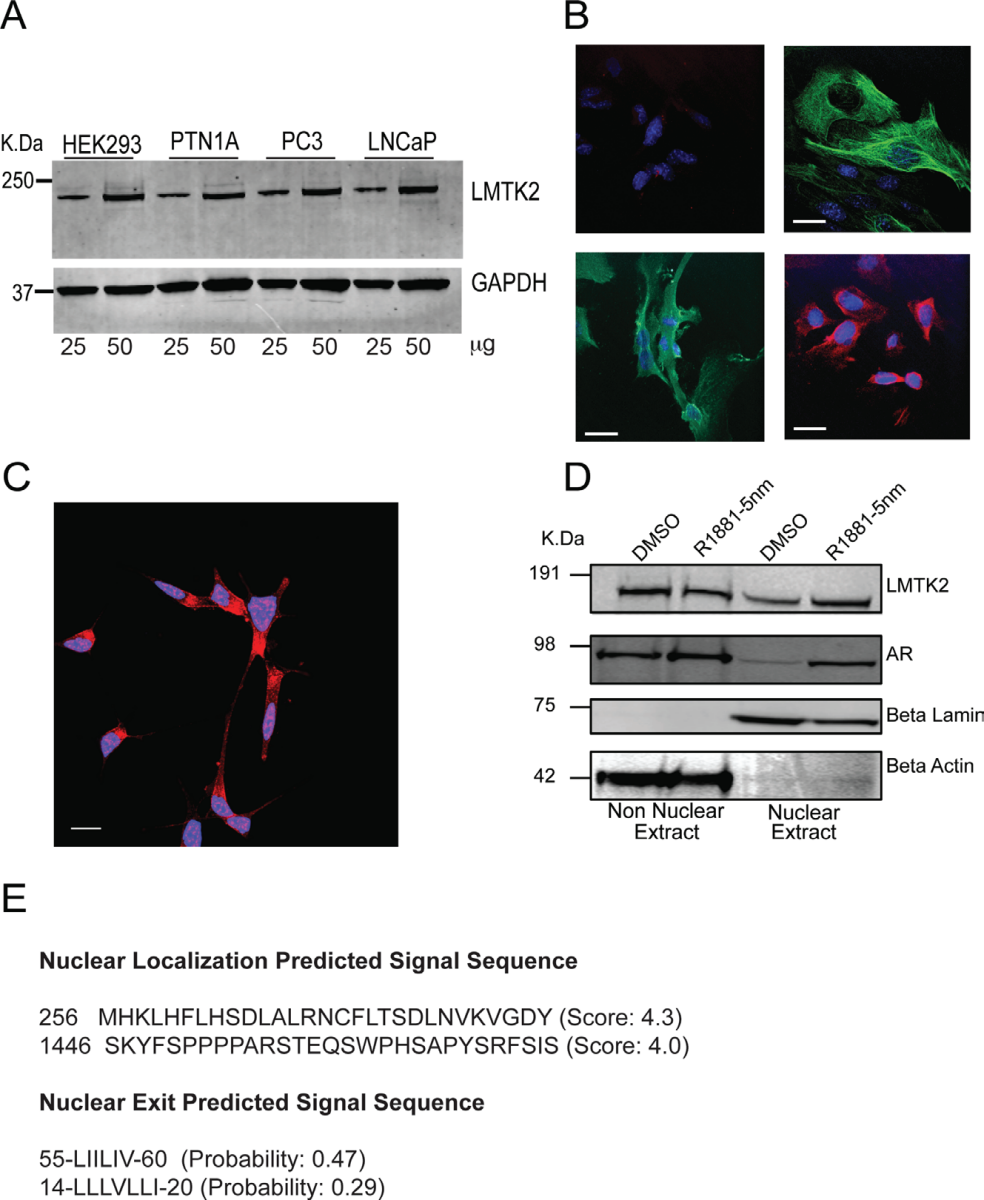
Expression and localization of *LMTK2* in prostate epithelial cells **A.** Immunoblot showing expression of *LMTK2* in Human Embryonic Kidney Cell (HEK293), Human prostate epithelial cell (PTN1A), Prostate Cancer cell (PC3) & Prostate adenocarcinoma cell (LNCaP). Two concentrations for each cell type were loaded **B.** Top-left panel shows the mice prostate epithelial cell stained with secondary antibody only; top-right panel shows mice prostate epithelial cell marker cytokeratin 5/8 stained with alexa 488 (Green). *LMTK2* is stained with alexa 488(Green) as shown in bottom left panel and bottom right panel shows the *AR* stained with CY-5 (Red). Nuclei stained in DAPI, appears blue. Bar = 0.25 μm. **C.** *LMTK2* stained with Cy5 (red) and nuclei are stained with DAPI (blue), colocalization between *LMTK2* and nuclei appears magenta. Bar = 0.5 μm. **D.** LNCaP cells grown in androgen deprived media were treated with DMSO or 2.5 nm R1881 (synthetic androgen) for 24 hours; cytoplasmic extract and nuclear extract were immunobloted to measure relative level of *LMTK2* and *AR*. Δ-lamin and Δ-actin were used as nuclear and cytoplasmic protein control, respectively. **E.** Predicted Multiple Putative NLS and NES by “cNLS Mapper” & NetNES 1.1 (http://www.cbs.dtu.dk/services/NetNES/) and ValidNESs (http://validness.ym.edu.tw/).

Given GWAS linking *LMTK2* expression levels with prostate cancer, we initially determined if *LMTK2* was expressed in prostate epithelia. We used a model cell line HEK293 as well as prostate cancer cell lines i.e. PTN1A, PC3 and LNCaP for the same. As predicted, immunoblot analysis showed robust endogenous expression of *LMTK2* in prostate epithelial and HEK293 cells, which appeared as a single dominant band of ~210 kDa (Fig. 2A), consistent with previously published data [26]. In addition, we confirmed that the observation were not an artifact of cell lines by studying *Lmtk2* expression in mouse primary prostate epithelial cells. Mouse primary prostate epithelial cells not only showed robust expression of *Cytokeratin* 5/8 (prostate epithelial cell marker) and AR as expected, but also *Lmtk2* (Fig. 2B).

Furthermore, several studies have showed *LMTK2* to be an endosome membrane-anchored protein [26, 34]. Hence, a reasonable expectation was that *LMTK2* would be localized in the extra-nuclear membrane fraction of prostate cancer cells. Surprisingly, our confocal images showed both nuclear as well as non-nuclear staining for *LMTK2* in prostate cancer cells (Fig. 2C). We further confirmed this finding using subcellular fractionation, to enrich a nuclear fraction, which too showed presence of *LMTK2* in nuclear and non-nuclear compartment of prostate cancer cells, irrespective of its androgen exposure (Fig. 2D). AR translocation, as reported in previous studies [37] was also seen in the fractionation analysis.

### *LMTK2* is down regulated in human prostate cancer

**Figure 3:**
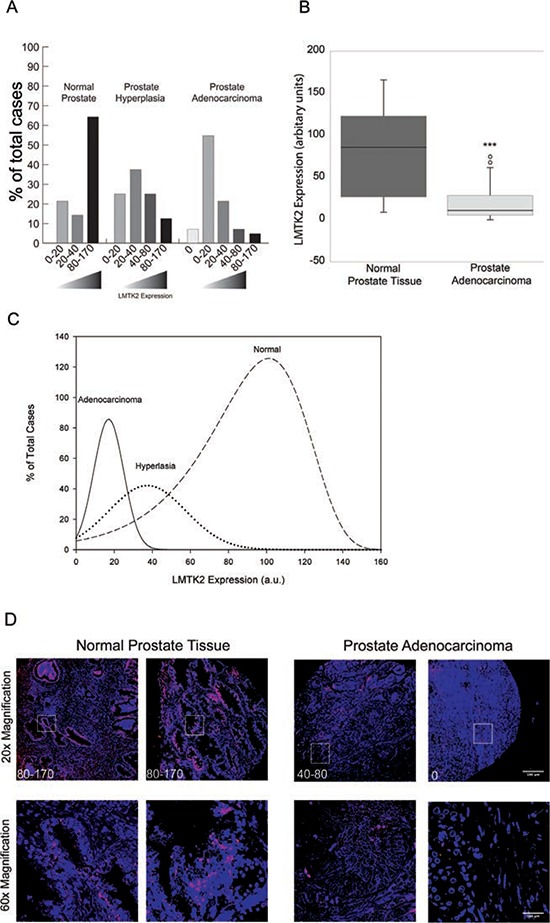
*LMTK2* is down regulated in human prostate cancer **A.** Expression levels of *LMTK2* protein in 48 prostate cancer, 8 prostate hyperplasia and 14 normal prostate tissue samples measured by tissue array. *LMTK2* protein levels were detected by immunofluorescence staining. Intensity of staining was classified as no (0), low (0–20), medium (20–40), high (40–80) and very high (80–170). **B.** This box plot gives the cancer status (Normal or prostate adenocarcinoma) on the X-axis, and the protein expression of *LMTK2* on the Y-axis. Error bars represent the interquartile range (IQR) of the measurements. The level of significance, *P* ≤ 0.001, was determined by Mann-Whitney-*U* analysis for pairwise comparison and circles indicate outliers. **C.** To generate curves, data were fit using a Best-Fit Gamma distribution (SigmaPlot, Systat, San Jose, CA). **D.** Representative Immunofluorescence images, *LMTK2* stained with secondary antibody linked to Cy-5 (red) and nuclei stained in DAPI. Top panel shows low magnification image, the boxed region is magnified in the bottom panel. Scale bars = 100 μm.

Previous studies have suggested that reduced *LMTK2* mRNA levels are associated with prostate cancer, however whether this translates to altered protein levels has not been determined. Immunostaining analysis of a human prostate tissue array (US Biomax) containing prostate cancer (*n* = 48), prostate hyperplasia (*n* = 8) and normal prostate tissue (*n* = 14) from a total of 20 individual patients, revealed a marked difference in *LMTK2* protein expression levels (Supplementary Table 2). *LMTK2* intensity was determined using Image-J software and assigned arbitrary unit, which was binned as no (0), low (0–20), medium (20–40), high (40–80) and very high (80–170). A majority, >65% of normal prostate tissue had very high expression of *LMTK2*, around 13% and 25% of hyperplasia tissue had respectively very high or high expression of *LMTK2*. In contrast, >67% of tumor tissue contained undetectable or low levels of *LMTK2* (Fig. 3A, 3C and 3D). The statistical significance of apparent differences in *LMTK2* expression between normal, and prostate cancer was investigated by Mann-Whitney-*U* analysis for pairwise comparison, which revealed a strong association (*P* ≤ 0.001) between a decrease in *LMTK2* protein expression and prostate cancer (Fig. 3B).

### *LMTK2* and AR interact in prostate cancer epithelial cells and co-localizes in human prostate tissue

**Figure 4:**
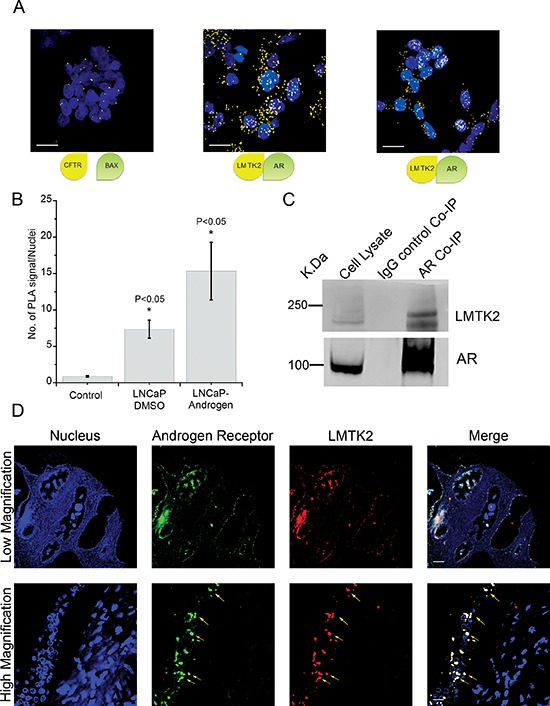
*LMTK2* and AR interact in prostate cancer epithelial cells and co-localizes in human prostate tissue **A.** Protein within interacting distance i.e. <40 nm is detected using proximity ligation assay (PLA). Cartoon shows two interacting protein being detected by fluorescent signal. PLA between non-interacting proteins, *CFTR* and *BAX* was used as negative control. PLA between *LMTK2* and *AR* in LNCaP cells grown in DMSO or 2.5 nM R1881 (androgen) shows PLA signal (Yellow dots), representing interaction. **B.** *LMTK2-AR* PLA signals per nucleus in LNCaP cells are counted using Image J software & compared with *CFTR-BAX* (non interacting proteins). Error bars indicate SD, which is given for mean of *n* ≥ 30 cells per condition. *P < 0.05 for difference from *CFTR-BAX* by student-unpaired *t*-test. **C.** Lysates from LNCaP cells was precipitated using mouse anti-AR Ab or control mouse Ab and blotted for *LMTK2* or AR. 10% of lysate was loaded in the first lane as lysate control. **D.** Representative figure for co-localization between *LMTK2* (red) and *AR* (green) in normal human prostate tissue is shown. Nuclei were stained with DAPI (blue). Arrows in the merge image shows some, but not all areas of co-localization (yellow) between *LMTK2* and *AR*. Top panel shows low magnification image, the boxed region is magnified in the bottom panel.

We had initially hypothesized that *LMTK2* might negatively regulate AR-dependent transcriptional activity. Hence, we asked whether *LMTK2* and AR are binding partners in prostate epithelial cells. Protein complexes immunoprecipitated with AR antibody from whole cell lysate of LNCaP cells indicated the presence of *LMTK2* (Fig. 4C). The specificity of *LMTK2* and AR interaction was shown by absence of *LMTK2* when AR antibody was replaced by IgG (Fig. 4C). AR pull down was confirmed by blotting with separate AR antibody (AR-N20) (Fig. 4C). However, in the reciprocal Co-IP experiment, *LMTK2* antibody failed to pull down AR, this might be due to *LMTK2* antibody interfering with the binding site to the AR.

In addition, a Proximity Ligation Assay (PLA), a means of assessing protein-protein interaction *in situ*, was used to verify CO-IP findings [38]. *LMTK2*/AR complexes were labeled using antibodies directed against *LMTK2* and AR in LNCaP cells deprived of androgen or grown in the presence of synthetic androgen (R1881, 2.5 nM). Significantly higher numbers of *LMTK2*/AR protein complexes were detected in prostate epithelial cells in comparison to negative control (*BAX*/*CFTR* complexes), two known non-interacting protein (Fig. 4A and 4B). While under androgen deprivation the distribution of the *LMTK2*/AR complexes was predominantly extra-nuclear but in presence of androgens, they also localized within the nuclear space.

To rule out cell line artifact, we further explored possible interaction of the AR and *LMTK2* in human prostate tissue using co-localization analysis. 16 normal prostate tissue samples were tested using immunostaining for possible co-localization between AR and *LMTK2*. Fig. 4D shows representative figure where AR and *LMTK2* appear to co-localize in the glandular prostate epithelial cells. Results obtained from human prostate cancer cell and tissue model together shows that AR and *LMTK2* are binding partners suggesting a functional significance of *LMTK2* in AR-axis.

### *LMTK2* inhibits AR transcriptional activity

**Figure 5:**
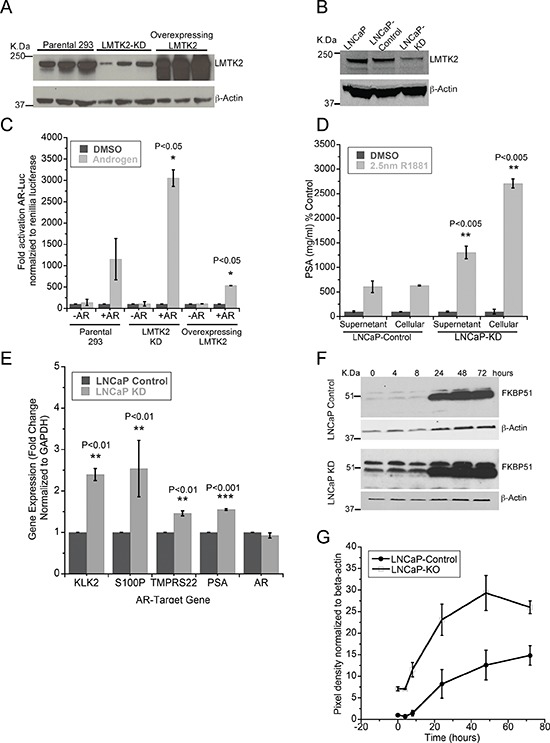
*LMTK2* inhibits AR transcriptional activity **A.** Immunoblot comparing *LMTK2* expression between HEK293 cells stably transfected with control shRNA (Parental 293), *LMTK2* shRNA (*LMTK2*-KD) or PCI-*LMTK2* (Overexpressing *LMTK2*). β-actin was used as endogenous loading control. **B.** Immunoblot comparing *LMTK2* expression between LNCaP cells and LNCaP cell stably transfected with either control shRNA (LNCaP-control) or *LMTK2* shRNA (LNCaP-KD). β-actin was used as endogenous loading control. **C.** Modulation of *AR* responsive gene activity by *LMTK2* (mean ± SD, *n* = 3) *p < 0.05 for difference from endogenous *LMTK2* levels in HEK293 cells. **D.** ELISA was performed in duplicates to analyze the concentrations of *PSA* protein in culture supernatant and cell lysate from LNCaP sublines cultured in androgen deprived medium for 3 days and treated with either DMSO or 2.5 nM synthetic androgen (R1881) for 16 hours (R1881) for 24 hours. Data is presented as % change in relation to the control (DMSO treated), (mean ± SD, *n* = 3) **p < 0.005 for difference from DMSO treatment group. **E.** Real-time PCR comparing transcript level of *AR* and genes regulated by *AR* between LNCaP sublines grown under androgen deprivation for 3 days was performed in duplicates (*KLK2*, *S100P*, *TMPRS22*, *PSA*), (mean ± SD, *n* = 3) **p < 0.01, ***p < 0.001 value for difference from endogenous *LMTK2* levels in LNCaP cells are denoted on the graph. **F, G.** Immunobloting of *FKBP51* in control and knock down cells grown under androgen deprivation for 3 days and treated with 1 nm R1881 for time points shown in figure. Quantitative analysis of normalized protein levels of *FKBP51* between control and knock down LNCaP cells is shown in panel **G**.

Our study shows that a decrease in *LMTK2* expression is associated with human prostate cancer and that *LMTK2* and AR are binding partners in prostate epithelial cells. These results prompted us to examine the potential role for *LMTK2* in AR-mediated signaling. For this purpose, we created HEK293 cells stably expressing either shRNA against *LMTK2*, control shRNA (scrambled sequence) or an *LMTK2* over-expression plasmid. We also created stable LNCaP cells expressing either shRNA against *LMTK2* (LNCaP-KD cells) or control shRNA (LNCaP-Control cells). Knock down or overexpression of *LMTK2* in these cell lines was confirmed using immunoblot analysis (Fig. 5A & 5B). To measure the affects of manipulating *LMTK2* level on the androgen receptor activity we used a dual luciferase assay. Overexpression of *LMTK2* in HEK293 cells expressing AR, decreased androgen-dependent activation of a luciferase reporter gene by two fold compared to parental cells expressing AR; no activation was observed in HEK293 cells without AR. In contrast, knock down of *LMTK2* in HEK293 cells expressing AR (analogous to prostate cancer cells) enhanced androgen-dependent activation of reporter gene by three fold in comparison to parental cells and by six fold in comparison to cells overexpressing *LMTK2* (Fig. 5C). Constitutively active renillia luciferase was used as transfection control.

To study the affects of *LMTK2* on the expression of endogeneous AR regulated genes, we measured the expression of *PSA*, a prototypical AR regulated gene [39]. Consistent with the luciferase assay in HEK293 cells, there was a dramatic increase in activation of endogenous AR in LNCaP-KD cells in comparison to LNCaP-Control as measured by secreted and total *PSA* (Fig. 5D).

### Decrease in *LMTK2* expression increases basal AR activity

Since, the AR is active in CRPC and is able to transcribe AR-dependent genes either in the presence of low levels of androgens or absence of androgens [40], we determined if a decrease in *LMTK2* is involved in activating AR in androgen deprived prostate cancer cells. Thus, we compared the expression of AR responsive genes between LNCaP-KD and LNCaP-Control cells deprived of androgens for 72 hours. *LMTK2*-KD cells showed a significant increase in mRNA expression of the AR dependent genes-*KLK2*, *S100P*, *TMPRS22* and *PSA* (Fig. 5E) in comparison with LNCaP-control cells, no significant difference in mRNA levels of AR was found. No significant differences were observed in the initial experiments using *GAPDH* or *Tubulin* as internal control; hence experiments were conducted using GAPDH as internal control. In addition, we also compared protein expression of *FKBP51*, an AR regulated gene, between LNCaP-KD and LNCaP-control cells grown in absence of androgen for 72 hours followed by androgen (1 nm R1881) stimulation for times shown in the Fig. 5F. We found that *LMTK2*-KD significantly increased *FKBP51* protein levels in comparison to LNCaP-control cells, with the biggest difference of about 5 fold was observed at time zero i.e. during the androgen deprivation stage (Fig. 5F and 5G).

### *LMTK2* down-regulation promotes tumor forming capacity and proliferation in LNCaP cells

**Figure 6:**
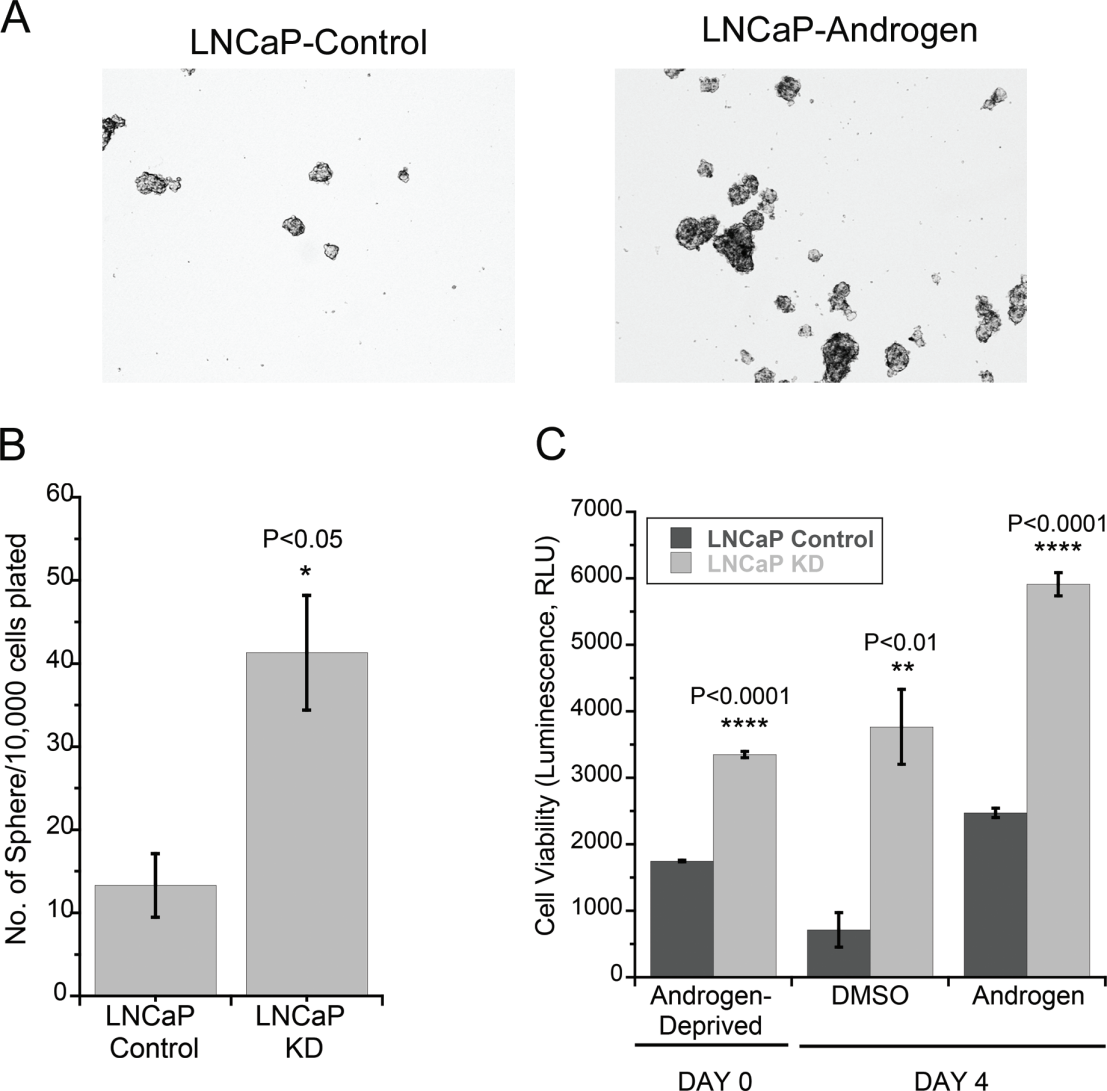
*LMTK2* down-regulation promotes tumor forming capacity and proliferation in LNCaP cells **A.** Panel showing affects of endogenous *LMTK2* levels on LNCaP tumor forming capacity. Representative colonies are shown in the figure. Quantitative analysis of colony numbers is shown in the panel **B.** (mean ± SD, *n* = 3) *p < 0.05 for difference from endogenous *LMTK2* in LNCaP cells. **C.** Androgen-independent and androgen-stimulated cell viability of LNCaP-control and LNCaP-KD cell lines measured by the ATP-Glo™ Bioluminometric cell viability assay, (mean ± SD, *n* = 3) ****p < 0001, **p < 0.01, ****p < 0.0001 value for difference from endogenous *LMTK2* in LNCaP cells.

In order to better understand the physiological role of *LMTK2* in prostate cancer, we investigated the effect of *LMTK2* on the tumor forming capacity and cell viability of LNCaP cells, using a tumorsphere assay [41] and a cell viability assay. Tumorspheres are enriched in cancer stem cells [42], which are argued to be tumor-initiating cells and are believed to play an enabling role in development of CRPC [43]. LNCaP-KD cells showed significantly higher colony-forming capacity by forming ~5 times more clones compared to LNCaP control cells in tumorsphere assay (Fig. 6A & 6B). We further tested the effects of a decrease in *LMTK2* expression on prostate cancer cell viability using the ATP-Glo Bioluminometric cell viability assay (Biotium, CA). LNCaP-KD cells, as expected, showed ~5 times higher cell viability under androgen starvation and ~2.5 times in presence of androgen when compared to LNCaP-control cells (Fig. 6C). It is important to note that while LNCaP-control cells treated with DMSO after 3 days of androgen starvation showed decrease in cell viability, there was no such decrease observed in LNCaP-KD cells.

## DISCUSSION

Recently, several Genome Wide Association Studies (GWAS) involving prostate cancer patients of Caucasian and East Asian descent identified a genetic variant (SNP rs6465657) of Lemur Tyrosine Kinase 2 (*LMTK2*), leading to decrease in its mRNA expression, to be associated with prostate cancer [29–32]. However, no information is available in terms of protein expression or function of *LMTK2* in prostate epithelial cells. In this report, we compared protein expression of *LMTK2* in human prostate cancer tissue specimens with normal, hyperplasia and prostate tissue specimens. Our data shows that loss of *LMTK2* protein is strongly associated with prostate cancer and prostate hyperplasia, disease states marked by dysregulation of AR. Since, we did not genotype prostate cancer tissues it remains to be determined if SNP rs6465657 associated with prostate cancer results in decrease in *LMTK2* protein expression. Furthermore, we also show a high-level expression of *LMTK2* in normal prostate epithelial cells (PNT1A), androgen-independent metastatic prostate cancer cells (PC3), androgen-dependent prostate cancer cells (LNCaP) and mouse primary prostate epithelial cells. Since, *LMTK2* was expressed in androgen dependent as well as androgen independent prostate cancer epithelial cells, it is likely that function of *LMTK2* is not just limited to AR axis and certainly previous studies have shown *LMTK2* to be an important binding partner of myosin VI involved in endocytic trafficking pathways in prostate cancer cells [33]. Furthermore, this report proposes a novel function for *LMTK2*, as a negative regulator of AR transcription.

It was surprising to find *LMTK2* to be localized in a nuclear fraction of prostate cancer cells as previous studies have suggested *LMTK2* to be an endosomal-membrane associated kinase [26, 34] though none looked specifically for nuclear localization. Using a Nuclear Localization Signal (NLS) prediction tool [44–46] and Nuclear Exit Signal (NES) prediction tool [47], we predict 2 possible bipartite NLS signal and a N-terminal NES signal in *LMTK2* (Fig. 2E), however these sites remain to be confirmed experimentally. Hence, we speculate that *LMTK2* might be involved in retrograde endocytic transport of proteins i.e. from cytoplasmic fraction to nuclear fraction. Furthermore, *LMTK2* immunostaining and duolink data in this report indicates a nuclear translocation of *LMTK2*/AR complexes in response to androgen treatment of prostate cancer cells. Together, these data suggests that *LMTK2* may be involved in the activation and translocation of AR. This phenomenon of an endosomal membrane protein localized in nuclear fractions is not unique to *LMTK2*. For example, *FAM21*, a WASH complex subunit residing in early endosome membrane like *LMTK2* has been shown to undergo nuclear translocation and participate in NF-kB-depenent gene regulation in pancreatic cancer cells [48, 49]. Similarly, tachykinin NK3 receptor (*NK3R*), a multi-pass membrane protein belonging to G-protein receptor-1 class undergoes nuclear translocation via importin pathway [50, 51].

The majority of prostate cancers (PCa) and Castrate Resistant Prostate Cancers (CRPC) have functional AR, which continues to drive the expression of AR-dependent cell proliferative genes and hence the tumor growth despite of low level of systemic androgens [52, 53]. Our report shows that *LMTK2* is an essential negative regulator of AR transcriptional activity and knocking down its expression in prostate cancer cells leads to significant increase in the AR activity and also an increase in tumorogenecity and cell viability. In contrast, overexpressing *LMTK2* leads to repression of AR activity. Hence, it can be deduced that a decrease in *LMTK2* expression observed in prostate cancer patient promotes tumor cells proliferation by enhancing AR transcriptional activity.

One of the most important outcomes from this report was the role of *LMTK2* in CRPC. Kinases play an important role in driving AR activity in CRPC either by direct activation of AR or by increasing its sensitivity to low level of androgens. *FKBP51*, an AR-dependent gene, which is also a positive regulator of AR activity, is expressed two fold higher in CRPC compared with primary tumors [54]. Data in this paper shows that decreasing *LMTK2* expression in prostate cancer cells deprived of androgen results in significantly higher levels of *FKBP51* protein as well as increased mRNA levels of AR-dependent genes (*KLK2*, *S100P*, *TMPRS22* and *PSA*). These results provided strong circumstantial evidence of a role for *LMTK2* in pathogenesis and progression of prostate cancer to castrate resistant stage. In addition, cell viability data showed the strongest role of decrease in *LMTK2* in regards to androgen-independent growth in prostate cancer cells, androgen-dependent growth was also affected, although to a lesser degree.

An important question that arises from our study is the mechanism by which *LMTK2* might be regulating AR transcription and cell proliferation in the absence of exogenous androgen in prostate cancer cells. There are several possible mechanisms that might be playing a role. Firstly, *LMTK2* has been shown to inactivate catalytic activity of *PP1*, which plays an important role in nuclear retention of AR by dephosphorylating at Ser-650. Hence a decrease in *LMTK2* protein express might result in increase in *PP1* activity leading to increased nuclear retention and hence increased transcription activity. Secondly, *LMTK2* might be directly phosphorylating AR and resulting in increased transcription activity in absence or presence of exogenous androgen. Initial evidence supporting this possibility comes from our study where we show *LMTK2* and AR to be binding partners in prostate cancer cells. However, further experiments are needed to support this hypothesis. And lastly, *FKBP51*, an AR regulated gene is also known to positively regulate AR transcriptional activity. Our study showed increase in *FKBP51* protein expression in *LMTK2*-KD prostate cancer cells. Argument can be made that *LMTK2* regulates AR activity through *FKBP51*. Further experiments are clearly needed to identify possible mechanism through which *LMTK2* augments AR transcription and cell proliferation in prostate cancer cells.

In conclusion, our findings are the first evidence that *LMTK2* negatively regulates AR activity in prostate cancer cells possibly by directly interacting with AR. Furthermore, loss of *LMTK2*, associated with prostate cancer can enhance AR transcriptional activity in absence of androgen, suggesting role of *LMTK2* in development of CRPC. *LMTK2* can now be considered negative regulator of androgen-induced AR-mediated cell growth and transcription. In terms of potential therapeutic target, small molecules that enhance the activity of *LMTK2* can decrease AR-proliferative activity in patients with prostate cancer and more importantly with castrate resistant prostate cancer.

## MATERIALS AND METHODS

### Cell culture

The human cell lines, PNT1A (normal prostate epithelial cells, Sigma-Aldrich, St. Louis, Mo) and LNCaP (androgen sensitive human prostate adenocarcinoma cells, ATCC, Manassas, VA) were cultured in RPMI 1640 medium while PC3 (metastatic prostate cancer cells isolated from bones, ATCC) were maintained in F-12K medium. HEK293 (Female-Human Embryonic Kidney Epithelial cells, ATCC) were cultured in Advanced Dulbecco's Medium. All culture medium were supplemented with 10% FBS and 1% Pen/Strep (Invitrogen, Carlsbad, CA) unless specified.

### Plasmids and transfections

Wild type *LMTK2* cloned in PCI vector was provided as a gift by Dr. Takeshi Inoue, University of Tokyo. Halo tagged Androgen Receptor construct was obtained from Promega (Madison, WI). Plasmids expressing and shRNA against human *LMTK2* or control shRNA were obtained from DNA 2.0 (Menlo Park, CA). Transfections of cells were performed by 4D nucleofector (Lonza Group Ltd, Basel, Switzerland) according to the manufacturer's instruction.

### Prostate primary cell isolation

Male mice were euthanized at 8–12 week of age and primary prostate epithelial cells were isolated as described [55]. Cell type was verified by staining for Cytokeratin 5/8 (SC-32328, Santa Cruz Biotechnology, Santa Cruz, CA), widely used marker for prostate epithelial cells [56].

### Immunofluorescence

Cells plated onto poly-L-lysine coated coverslips were fixed according to pH-shift protocol as described [57] and stained for AR (AR441, Santa Cruz Biotechnology) and *LMTK2* (HPA010657, Sigma-Aldrich). Coverslips were mounted onto a slide using Prolong Gold with DAPI (Invitrogen). The cellular signal was visualized using a PlanApo 60 ×, 1.42 NA oil immersion objective of an Olympus IX71 inverted microscope (Olympus, Center Valley, PA) coupled to a VT-Infinity 3 confocal system (VisiTech International, Sunderland, UK). For each sample, multiple coverslips were imaged (≥30 cells per coverslip) under identical settings.

### Fractionation of membrane/cytoplasmic and nuclear proteins

LNCaP cells were starved of androgen for 3 days by replacing complete media containing FBS with Charcoal Stripped Fetal Bovine Serum (CSFBS). On day 3, cells were treated either with DMSO or R1881 (Sigma-Aldrich) as described in results. Nuclear and non-nuclear fractions were separated as per manufacture's recommendation (GeneTex, Inc., Irvine, CA) and were probed for *LMTK2* and AR using Immunoblot analyses. Anti-human GAPDH antibody (SC-25778) was used for analyzing non-nuclear fraction while Lamin-A (SC-20680) was used for nuclear fraction.

### Co-immunoprecipitation and immunoblot analyses

LNCaP cells were lysed in 1% NP-40 lysis buffer and endogenous complexes of AR were immunoprecipitated using mouse anti-AR 441 antibody. Immunprecipitated complexes were then probed for *LMTK2* (rabbit anti-LMTK2) using immunoblot assay. AR pull down was confirmed by blotting with rabbit anti-AR N20 antibody. For immunoblot analyses samples were prepared in 4 × LDS sample buffer, resolved on 4–12% gradient Tris-Glycine Gel and transferred on to polyvinyldiflouride (PVDF) membrane. Membranes were probed for several proteins using human anti-AR, anti-*LMTK2*, anti-β-*ACTIN* (LI-COR Biotechnology, Lincoln, NE) and anti-*FKBP51* (ab-2901, ABCAM, Cambridge, MA) antibodies. Proteins were detected and analyzed using Odyssey Sa-Infrared imaging system™ (Li-COR Biotechnology).

### Tissue immunohistochemistry

Tissue arrays (BC19021a) obtained from US-Biomax (Rockville, MD) containing normal, malignant and metastatic human prostate tissue was used. The arrays were probed for *LMTK2* and AR using a Tyramide Signal Amplification Kit (TSA, Invitrogen) as per manufacturer's recommendation.

### Proximity ligation assay

A Proximity Ligation Assay kit (Olink Bioscience, Uppsala, Sweden) was used to study the interaction between AR and *LMTK2* in LNCaP cells [58]. Staining procedure were carried out following the manufacturer's instructions using rabbit anti-*LMTK2* and mouse anti-AR antibodies to detect AR/*LMTK2* interactions. *BAX* (Bcl2 associated protein-X) and *CFTR* (Cystic Fibrosis Trans membrane Conductance Regulator) are non-interacting proteins, and hence rabbit anti-*BAX* (sc-493) and mouse anti-*CFTR* (596, UNC at Chapel Hill, NC) were used as negative control. The cellular PLA signal was visualized using the Olympus IX71 inverted microscope as described above and quantified using Image-J software [59].

### Real-Time RT-PCR

Total cellular RNA was extracted from cells using the Trizol RNA isolation reagent according to manufacturer's instruction (Invitrogen). Complementary DNA synthesis reactions were performed with 1 μg of RNA using SuperScript^®^ III First-Strand Synthesis System (Invitrogen) according to manufacturer instruction. cDNA samples were amplified using SYBR^®^ Green PCR Master Mix on the Applied Biosystems 7500 Detection System. Gene-specific forward and reverse primers (Supplementary Table 1) used have been reported in earlier studies [60, 61]. Furthermore, specificity and efficiency for primers were analyzed by running qPCR with series of cDNA dilutions and specific amplification for every assay were confirmed by melt curve analysis. All assays were run in duplicates and were repeated 3 times. The amplified transcripts were quantified using the comparative **ΔΔ^Ct^** method.

### PSA measurement

*PSA* (Prostate Specific Antigen) protein levels were detected using an enzyme-linked immunosorbent assay (ELISA) technique [62]. Cells were plated into 96-well plates at density of 1 × 10^5^ per well in RPMI media supplemented with 10% CSFBS. After 3 days, cells were treated for 16 h as detailed in results. *PSA* in the cultural supernatant and cellular *PSA* was quantified with an ELISA kit (Abcam) following the manufacturer's instruction. All assays were run in duplicates and repeated 3 times.

### Dual luciferase assay

Cells (1 × 10^5^ per well in 96-well plates) were co-transfected with Halo-AR and/or either AR reporter, negative control or positive control in 2:1 ratio, supplied with cignal androgen receptor reporter kit (Qiagen, Valencia, CA). Cells were grown in charcoal-stripped media for 2 days and were treated with 1nm R1881 for 16 h. Luciferase activities were measured by using the dual-luciferase reporter gene assay system (Promega, Madison, WI) following the manufacturer's instruction in plate reader (POLARstar Omega, BMG Labtech, Germany). Final results were normalized for transfection efficiencies using the Renillia Luciferase Assay Value. Luciferase assay were repeated for 3 times.

### Cell viability assays

1 × 10^5^ cells were plated per well onto flat clear bottom white polystyrene 96-well plate (Nunc) in RPMI media supplemented with 10% CSFBS. Cells were treated as discussed in the result section. Cell viability was measured using ATP-Glo^TM^ Bioluminometric cell viability assay kit (Biotium, CA.) according to manufacturer's instruction [63]. Each assay was performed in triplicate and repeated 3 times (*n* = 3). Luminescence was measured using POLARstar Omega.

### Tumorigenicity assay

Cells were harvested and resulting pellet was washed in PBS. 1 × 10^5^ cells per well were plated onto 6-well Ultra-Low Attachment Plates (Corning, NY) in Tumorsphere medium (PrEGM media with supplied growth factors and supplements+1% N2 (Invitrogen) and 1% B27 (Invitrogen). After 7 days number of spheroids (solid, rounded structures) were counted under light microscope. Each experiment was carried out in triplicates.

## SUPPLEMENTARY TABLES


