# Supplementary material for: Lemur Tyrosine Kinase 2, a novel target in prostate cancer therapy
Source: Oncotarget. 2015 May 8;6(16):14233–46. doi: 10.18632/oncotarget.3899 (PMC4546463; doi:10.18632/oncotarget.3899)
Supplement: Supplementary file 1 [file oncotarget-06-14233-s001.pdf]

SUPPLEMENTARY TABLES

Supplementary Table S1. (Real time PCR Primers)

1	KLK2-F	5′-CAC AGC TGC CCA TTG CCT AAA GAA-3′
	KLK2-R	5′-GGC CTG TGT CTT CAG GCT CAA A-3′
2	S100P-F	5′-ATG ACG GAA CTA GAG ACA GCC-3′
	S100P-R	5′-AGG AAG CCT GGT AGC TCC TT-3′
3	TMPRSS2-F	5′-GGA CAG TGT GCA CCT CAA AGA C-3′
	TMPRSS2-R	5′-TCC CAC GAG GAA GGT CCC-3′
4	PSA-F	5′-TGG TGC ATT ACC GGA AAG TGG ATC A-3′
	PSA-R	5′-GCT TGA GTC TTG GCC TGG TCA TTT C-3′
5	AR-F	5′-TCT TGT CGT CTT CGG AAA TGT-3′
	AR-R	5′-AAG CCT CTC CTT CCT CCT GTA-3′
6	GAPDH-F	5′-GGAAGGTGAAGGTCGGAGTC-3′
	GAPDH-R	5′CTGGAAGATGGTGATGGGATTTC-3′

Supplementary Table S2.
Catalog #: BC19021 (Biomax)
Description: Prostate carcinoma, adjacent normal tissue and normal tissue array
Lot #: BC19021
Total cases: Twenty (20) individual patients
Total array dots: Seventy-two (72)
Array panel: This is a COMBINATION ARRAY PANEL from diseased, normal and adjacent (1.5 cm) to specimen that was selected and pathologically confirmed
Array dot diameter: 1.5 mm
Section thickness: 5 micrometers

No.	Age	Organ	Pathology diagnosis	Grade	TNM	Gleason score	LMTK2 Expression Value (a.u)
01	62	Prostate	Adenocarcinoma	II	T3N1M1b	3+3	11
02	62	Prostate	Adenocarcinoma	II	T3N1M1b	3+3	18
03	62	Prostate	Adenocarcinoma	II	T3N1M1b	3+3	52
04	75	Prostate	Adenocarcinoma	I	T2N1M1c	1+1	7
05	75	Prostate	Adenocarcinoma	I	T2N1M1c	1+1	3
06	75	Prostate	Adenocarcinoma	I	T2N1M1c	1+1	9
07	75	Prostate	Prostate tissue	-	-	-	17
08	75	Prostate	Prostate tissue	-	-	-	17
09	82	Prostate	Adenocarcinoma	III	T2N0M0	5+5	5
10	82	Prostate	Adenocarcinoma	IV	T2N0M0	5+5	28
11	82	Prostate	Adenocarcinoma	IV	T2N0M0	5+5	7
12	40	Prostate	Adenocarcinoma	IV	T2N1M1b	4+4	9
13	40	Prostate	Adenocarcinoma	IV	T2N1M1b	4+4	40
14	40	Prostate	Adenocarcinoma	IV	T2N1M1b	4+4	28
15	40	Prostate	Prostate tissue	-	-	-	166
16	40	Prostate	Prostate tissue	-	-	-	36
17	21	Prostate	Prostate tissue	-	-	-	11
18	21	Prostate	Smooth muscle tissue	-	-	-	9
19	72	Prostate	Adenocarcinoma	IV	T2NxM0	5+5	0
20	72	Prostate	Adenocarcinoma	IV	T2NxM0	5+5	26
21	72	Prostate	Adenocarcinoma	IV	T2NxM0	5+5	62
22	51	Prostate	Adenocarcinoma	II	T2N0M0	4+3	37
23	51	Prostate	Adenocarcinoma	II	T2N0M0	4+3	18
24	51	Prostate	Adenocarcinoma	II	T2N0M0	4+3	3
25	51	Prostate	Prostate tissue	-	-	-	150
26	51	Prostate	A little adenocarcinoma	IV	T2N0M0		75

(*Continued*)

No.	Age	Organ	Pathology diagnosis	Grade	TNM	Gleason score	LMTK2 Expression Value (a.u)
27	60	Prostate	Adenocarcinoma	IV	T2N0M0	5+5	3
28	60	Prostate	Adenocarcinoma	IV	T2N0M0	5+5	27
29	60	Prostate	Adenocarcinoma	IV	T2N0M0	5+5	8
30	66	Prostate	A little adenocarcinoma	I	T1N0M0	1+2	22
31	66	Prostate	A little adenocarcinoma	I	T1N0M0	1+2	14
32	66	Prostate	A little adenocarcinoma	I	T1N0M0	1+2	5
33	66	Prostate	Hyperplasia	-	-	-	115
34	66	Prostate	Hyperplasia	-	-	-	43
35	43	Prostate	Prostate tissue	-	-	-	159
36	43	Prostate	Prostate tissue	-	-	-	142
37	87	Prostate	Adenocarcinoma	IV	T2N0M0	5+5	10
38	87	Prostate	Adenocarcinoma	IV	T2N0M0	5+5	7
39	87	Prostate	Adenocarcinoma	IV	T2N0M0	5+5	11
40	69	Prostate	Smooth muscle tissue	-	-	-	66
41	69	Prostate	Smooth muscle tissue	-	-	-	97
42	69	Prostate	A little adenocarcinoma	IV	T4N0M0	4+5	68
43	69	Prostate	Hyperplasia	-	-	-	41
44	69	Prostate	Hyperplasia	-	-	-	29
45	63	Prostate	Adenocarcinoma	III	T2N1M1b	4+4	62
46	63	Prostate	Adenocarcinoma	III	T2N1M1b	4+4	4
47	63	Prostate	Adenocarcinoma	III	T2N1M1b	4+4	0
48	75	Prostate	Adenocarcinoma	IV	T3N0M0	5+5	33
49	75	Prostate	Adenocarcinoma	IV	T3N0M0	5+5	0
50	75	Prostate	Adenocarcinoma	IV	T3N0M0	5+5	11
51	75	Prostate	Hyperplasia	-	-	-	21
52	75	Prostate	Hyperplasia	-	-	-	25
53	25	Prostate	Prostate tissue	-	-	-	75
54	25	Prostate	Prostate tissue	-	-	-	115
55	66	Prostate	Adenocarcinoma	IV	T2N0M0	5+5	6

(*Continued*)

No.	Age	Organ	Pathology diagnosis	Grade	TNM	Gleason score	LMTK2 Expression Value (a.u)
56	66	Prostate	Adenocarcinoma	IV	T2N0M0	5+5	0
57	66	Prostate	Adenocarcinoma	IV	T2N0M0	5+5	1
58	55	Prostate	Adenocarcinoma	I	T2N0M0	2+2	13
59	55	Prostate	Adenocarcinoma	I	T2N0M0	2+2	9
60	55	Prostate	Adenocarcinoma	I	T2N0M0	2+2	0
61	55	Prostate	Prostate tissue	-	-	-	27
62	55	Prostate	Prostate tissue	-	-	-	106
63	62	Prostate	Adenocarcinoma	IV	T2N0M0	5+5	6
64	62	Prostate	Adenocarcinoma	IV	T2N0M0	5+5	51
65	62	Prostate	Adenocarcinoma	IV	T2N0M0	5+5	11
66	73	Prostate	Adenocarcinoma	II	T3N0M1b	2+2	30
67	73	Prostate	Adenocarcinoma	II	T3N0M1b	2+2	31
68	73	Prostate	Adenocarcinoma	II	T3N0M1b	2+2	50
69	73	Prostate	Hyperplasia	-	-	-	8
70	73	Prostate	Hyperplasia	-	-	-	12
71	27	Prostate	Prostate tissue	-	-	-	86
72	27	Prostate	Prostate tissue	-	-	-	123
